# The inevitable contrast: Conscious vs. unconscious processes in action control

**DOI:** 10.3389/fpsyg.2013.00590

**Published:** 2013-09-10

**Authors:** Ezequiel Morsella, T. Andrew Poehlman

**Affiliations:** ^1^Department of Psychology, San Francisco State UniversitySan Francisco, CA, USA; ^2^Department of Neurology, University of CaliforniaSan Francisco, San Francisco, CA, USA; ^3^Marketing Department, Cox School of Business, Southern Methodist UniversityDallas, TX, USA

**Keywords:** action, consciousness, unconscious processing, voluntary action, perception-and-action

The simple actions of everyday life—flicking a light switch, suppressing the urge to say something, or grabbing a waiter's attention with a “check, please”—remain difficult to understand from a scientific point of view. Unlike the mechanisms giving rise to machine action—which are designed according to clear-cut, well principled plans—the mechanisms underlying human action are fashioned by the happenstance and tinkering process of evolution, whose products can be counterintuitive and suboptimal (Simpson, 1949; Lorenz, 1963; Gould, 1977; de Waal, 2002; Marcus, 2008), far unlike the kinds of things we humans design into robots (Arkin, 1998)^1^. When speaking about the *reverse engineering* of biological products, the roboticist thus cautions, “Biological systems bring a large amount of evolutionary baggage unnecessary to support intelligent behavior in their silicon based counterparts” (Arkin, 1998, p. 32), and, speaking of the products of mother nature, the ethologist concludes, “To the biologist who knows the ways in which selection works and who is also aware of its limitations it is no way surprising to find, in its constructions, some details which are unnecessary or even detrimental to survival” (Lorenz, 1963, p. 260).

Faced with this and many other challenges (cf., Rosenbaum, 2005; Herwig et al., 2013), the student of human action is forced to abandon a *normative* view (which describes how things *should* function) of the phenomena at hand and adopt instead a more humble, *descriptive* view (which describes the products of nature as they have evolved to be). From such a descriptive approach, investigators over the past two decades have begun to illuminate, not only the basic processes underlying human action, but the liaison between action and consciousness—the most mysterious aspect of nervous function (Roach, 2005).

In this special issue of *Frontiers in Cognition*, we survey these advances stemming from disparate fields of inquiry, including cognition, neuroscience, and artificial intelligence/robotics. Together, these developments unveil a great deal about the links between perception and action while also illuminating much about all else in between. Of note, these developments also reveal that the study of *action production and control* (“action control,” for short) provides a unique portal through which to examine the nature of conscious processing. As explained below, many aspects of consciousness are easier to study from an *action-based approach* than from a *perception-based perspective*, which has been the traditional approach to studying consciousness (e.g., Crick and Koch, 2003; see discussion in Baars, 1997).

Before discussing further the liaison between consciousness and action control, and what the latter informs about the former, it is important to first describe the most nebulous term at hand, “consciousness.”

## The mind-boggling and (unfortunately) inescapable problem of consciousness and the brain

Throughout intellectual history, people have been investigating the phenomenon of consciousness in one way or another, though often while avoiding utterance of the controversial term, “consciousness,” which has been considered unscientific for most of its history. During the Behaviorist era (1919–1948), in which discussion of consciousness was strongly discouraged, the rank and file psychophysicist and Gestalt psychologist continued to study the “conscious field” that had been the object of investigation during the earlier Structuralist era pioneered by Wundt and Titchener (1879–1919). Since the fall of Behaviorism, a *de facto* distinction has been made between conscious and unconscious processing in every field of inquiry of psychology and neuroscience, though, again, often without mention of the term “consciousness.” In perception research, psychophysical measurement continues to make the distinction of *supra*- vs. *sub*liminal, and to base its conclusions on conscious “self-report.” In the study of attention, the term “attentional awareness” is often contrasted with unconscious, “pre-attentive” processing (Treisman and Gelade, 1980). In memory research, there is the classic distinction between “declarative” (explicit) processes and “procedural” (implicit) processes (Squire, 1987; Schacter, 1996). In research on motor control and on language production, the conscious aspects of voluntary action and action monitoring are contrasted with the unconscious aspects of motor programming (Levelt, 1989; Rosenbaum, 2002), including the implicit learning of motor sequences (Taylor and Ivry, 2013). Last, various fields contrast “controlled” processing, which tends to be associated with consciousness, and “automatic” processing, which tends to be associated with unconscious mechanisms (e.g., Lieberman, 2007; but see Panagiotaropoulos et al., 2013).

In summary, the difference between conscious and unconscious processes (regardless of the appellations ascribed to each process) is an inescapable contrast that is encountered after even a cursory examination of mental and nervous phenomena^2^.

Upon accepting that, in the natural world, there are conscious and unconscious processes, then one must contemplate the phenomenon of consciousness. Understanding how the nervous system gives rise to basic, low-level consciousness—the subjective experience of pain, breathlessness, or a yellow afterimage—remains one of the greatest puzzles in science (Crick, 1995; Roach, 2005). This most basic form of consciousness is referred to as “sentience” (Pinker, 1997), “subjective experience,” “phenomenal state,” and “qualia” (Gray, 2004). It has been best defined by Nagel (1974), who proposed that an organism possesses consciousness if there is *something it is like* to be that organism—something it is like, for example, to be human and experience pain, yellow afterimages, or breathlessness.

Some have attempted to *explain away* this mind-boggling puzzle by claiming that consciousness does not exist (which is perhaps the least deniable fact of our existence, given that consciousness encompasses the totality of all we know) or that it exists but serves no function (that is, it is “epiphenomenal”) in the nervous system. Unfortunately, while the former view is difficult to defend, the latter view does not provide an escape from the enigma at hand either. Regardless of whether consciousness serves a function in the nervous system or not, the scientist must still explain its place within nature: Huxley's steam whistle may be epiphenomenal with respect to the locomotive, but the scientist must still understand what it is (high frequencies) and how it arises from physical events (high pressured steam released through a small aperture). It seems premature to state that a phenomenon does not serve a function when the place of that phenomenon within nature remains unknown. In short, even if a phenomenon is functionless, a complete scientific account of the natural world must include an explication of it. See, in this issue, the article by Pereira et al. for a novel, untraditional approach to consciousness; see also relevant articles by Cruse and Schilling, by Hommel, and by Masicampo and Baumeister.

Progress regarding the puzzle of consciousness has stemmed from descriptive approaches juxtaposing conscious and unconscious processing in terms of their cognitive and neural correlates (Shallice, 1972; Logothetis and Schall, 1989; Crick and Koch, 1995; Kinsbourne, 1996; Wegner and Bargh, 1998; Grossberg, 1999; Di Lollo et al., 2000; Dehaene and Naccache, 2001; Baars, 2002, 2005; Gray, 2004; Libet, 2004; Laureys, 2005; Morsella, 2005; Merker, 2007; Doesburg et al., 2009; Damasio, 2010; Boly et al., 2011; Panagiotaropoulos et al., 2012). [For a review regarding the conclusions of this contrast, see Godwin et al. (2013); for discussion of the limitations of a contrastive approach, see Aru et al. (2012).] To examine this contrast, researchers have focused primarily on perceptual processing (see Panagiotaropoulos et al., 2013), for several important reasons (see reasons in Crick and Koch, 2003). Perception-based research has illuminated how entry into consciousness (“entry,” for short) is influenced by processes that are “bottom-up” (e.g., stimulus salience, motion, novelty, incentive and emotional quality, etc.; Gazzaley and D'Esposito, 2007) or attentional (cf., Most et al., 2005). This important research has led to several advances (see review in Koch, 2004), including (a) the differences in the processing of stimuli that are supraliminal (i.e., consciously-perceptible) and subliminal (i.e., consciously-imperceptible; Logothetis and Schall, 1989; Dehaene and Naccache, 2001; Koch, 2004; Roser and Gazzaniga, 2004; Doesburg et al., 2009), and (b) uncovering the unconscious processes preceding a conscious percept (Di Lollo et al., 2000; Goodhew et al., 2012; see Fischer et al., 2013).

Such research has also led to the *integration consensus* (Tononi and Edelman, 1988; Baars, 1988, 1998, 2005, 2013; Damasio, 1989; Freeman, 1991; Srinivasan et al., 1999; Zeki and Bartels, 1999; Edelman and Tononi, 2000; Dehaene and Naccache, 2001; Llinás and Ribary, 2001; Varela et al., 2001; Clark, 2002; Ortinski and Meador, 2004; Sergent and Dehaene, 2004; Morsella, 2005; Del Cul et al., 2007; Kriegel, 2007; Merker, 2007; Doesburg et al., 2009; Uhlhaas et al., 2009; Boly et al., 2011; Koch, 2012; Tallon-Baudry, 2012; Tononi, 2012), which proposes that consciousness integrates neural activities and information-processing structures that would otherwise be independent (see reviews in Baars, 2002; see Morsella, 2005, for the limitations of the integration consensus and for a listing of integrations that can occur unconsciously). Findings from action-based research complement the integration consensus: Consistent with the integration consensus, in conditions in which actions are decoupled from consciousness (e.g., in neurological disorders), actions often appear impulsive or inappropriate, as if they are not adequately influenced by the kinds of information by which they should be influenced (Morsella and Bargh, 2011). These actions reveal a lack of adequate integration. Thus, consciousness appears to permit a form of integration that constrains potential action, achieving a form of *multiple-constraint satisfaction* (Merker, 2013). Constraints can be “online,” reflecting stimuli in the current environment, or they can be “offline,” reflecting covert processes such as memory, cognitive maps, operations on mental representations, and mental simulation (Schacter and Addis, 2007). For example, recent theories propose that the function of explicit, episodic memory—a form of knowledge representation intimately associated with the past—is actually to simulate future, potential actions (Schacter and Addis, 2007).

## Consciousness and action

Although theorists have long appreciated that consciousness is intimately related to action (James, 1890; Neumann, 1987; Allport, 1989; Hamker, 2003; Morsella, 2005; Baddeley, 2007), until recently there has been a substantial gap in our knowledge regarding how action-related processes influence consciousness. The reason for this gap is not surprising, as action itself is an under-explored topic of research (see reasons for this in Nattkemper and Ziessler, 2004; Rosenbaum, 2005; Agnew et al., 2009; Herwig et al., 2013). Action control is a highly complicated process, one involving various kinds of mechanisms (e.g., *hierarchical* vs. *distributed control* and *forward modeling* vs. *inverse modeling*; Arkin, 1998; Miall, 2003). See in this issue, the article by Jordan. Only recently have researchers begun to focus on the action-related aspects of consciousness (e.g., Frith et al., 2000; Lau et al., 2004; Libet, 2004; Morsella, 2005; Berti and Pia, 2006; Jeannerod, 2006; Pacherie, 2008; Morsella and Bargh, 2010).

The following sections summarize those findings from action-based research that are relevant to this special issue about consciousness and action control (for a review of all action research, see Morsella, 2009)^3^.

### Unconscious processing in action control

Investigations on consciousness and action control have revealed that many sophisticated aspects of action production can or do occur unconsciously (Bargh and Morsella, 2008; Morsella and Bargh, 2011; see Panagiotaropoulos et al., 2013). Specifically, investigations from diverse areas (see review in Morsella and Bargh, 2011), including motor control (Rosenbaum, 2002), subliminal processing (Hallett, 2007), automatisms (Morsella and Bargh, 2011), dissociations between action and conscious perception (Goodale and Milner, 2004), and the automatic activation of action plans (Morsella and Miozzo, 2002; Ellis, 2009), reveal that the activation, modulation, selection, and, in some cases, expression of action plans can occur unconsciously. For example, research on various neurological conditions has revealed aspects of action control that can occur unconsciously. These neurological conditions include *blindsight* (Weiskrantz, 1992, 1997), *blind smell* (Sobel et al., 1999), *utilization behavior* (Lhermitte, 1983), *visual form agnosia* (e.g., Patient D. F.; Milner and Goodale, 1995), *anarchic hand syndrome* (Marchetti and Della Sala, 1998), *sensory neglect* (Graziano, 2001; Heilman et al., 2003), unintentional *ambient echolalia* (Suzuki et al., 2012), and complex automatisms, (e.g., vocalizations and singing) during epileptic seizures (Blanken et al., 1990; Enatsu et al., 2011; Kececi et al., 2013). Insights about consciousness and action control stemmed also from the study of the “split brain” patient (Sperry, 1961), and from conditions in which declarative memory is compromised but action programs can be stored and influence action even when the patient is unaware of the acquisition or maintenance of these programs (e.g., as in the case of H. M.; Milner, 1966). Together, this research provided substantial knowledge about the sophisticated capacities of unconscious processing in action control (see, in this issue, contributions by Cruse and Schilling, by Fischer et al., by Hommel, by Masicampo and Baumeister, by Panagiotaropoulos et al., and by Merker).

This research also reveals which aspects of action control may be unconscious during normal, everyday action, in which conscious and unconscious processes interact in ways that are only now beginning to be understood (see, in this issue, articles by Lynn et al., by Panagiotaropoulos et al., and by Merker). For instance, under normal circumstances, a person is unconscious of the complicated motor programs that, during action production, calculate which muscles should be activated at a given time (James, 1890; Rosenbaum, 2002; Johnson and Haggard, 2005; see Grossberg, 1999, about why motor programs must be unconscious). Specifically, evidence suggests that one is unconscious of the programming of the efference to the muscles as well as of the adjustments that are made “online” as one, say, reaches for a moving object (Fecteau et al., 2001; Rossetti, 2001; Rosenbaum, 2002; Goodale and Milner, 2004; Heath et al., 2008; Liu et al., 2008; see, in this issue, articles by Anderson et al. and by Rosenbaum et al.).

The activation of action plans (a phenomenon to be distinguished from motor control) can occur unintentionally (see Lynn et al., this issue). This has been revealed in experimental paradigms in which the mere presence of incidental action-related stimuli can interfere with one's intended response to a target stimulus. A basic form of this effect has been demonstrated for decades in the classic Stroop task (Stroop, 1935; see reviews in MacLeod and Dunbar, 1988; MacLeod, 1991; MacLeod and MacDonald, 2000), in which the mere presence of a word (e.g., RED) interferes with naming a patch of color (e.g., blue). In this task, participants are instructed to name the color in which a word is written. When the color matches the word (e.g., RED presented in red), or is presented on a neutral stimulus (e.g., a series of x's as in XXXX), there is little or no interference [e.g., decreased response times (RTs)] and decreased perturbations in consciousness (e.g., “urges to make a mistake”; Morsella et al., 2009a). (Urges to err, a *subjective effect*, are obtained simply by asking participants after each trial, “How strong was your urge to make a mistake?” which participants rate on an 8-point scale, in which 1 signifies “almost no urge” and 8 signifies “extremely strong urge.”) When the word and color are incongruous (e.g., RED presented in blue), response conflict leads to interference (Cohen et al., 1990), including increased RTs, error rates, and systematic changes in consciousness, such as urges to err (Morsella et al., 2009a).

In the incongruent condition, set-related top-down activation from prefrontal cortex increases the activation of areas in posterior brain regions (e.g., visual association cortex) that are associated with task-relevant dimensions (e.g., color; Enger and Hirsch, 2005; Gazzaley et al., 2005). Thus, to influence behavior, action sets from information in working memory or long-term memory increase or decrease the strength of perceptuosemantic information, along with, most likely, other kinds of information (e.g., motor priming). The finding that top-down activation strengthens one representation (e.g., color-naming) over another (e.g., word-reading) can be characterized as a case of “refreshing,” the act of foregrounding one representation over another (Johnson and Johnson, 2009). Following an incongruent trial, ramped up activation in control regions of the brain (e.g., the dorsolateral prefrontal cortex) leads to improved performance on the subsequent trial (Cohen et al., 1990).

### Paradigms illuminating the liaison between consciousness and action control

The Stroop task is one of many *response interference paradigms* (see, in this issue, articles by Anguera et al. and by Lynn et al.). In such paradigms, subjects attempt to respond to a *target* (e.g., font color in the Stroop task) while presented with a *distracto*r (e.g., Stroop word). Such interference paradigms have revealed much about the role of consciousness in action control. Findings complementing that of the Stroop paradigm have been obtained with the classic Eriksen flanker task (Eriksen and Eriksen, 1974). In one version of the task (Eriksen and Schultz, 1979), participants are trained to press one button with one finger when presented with the letter S or M and to press another button with another finger when presented with the letter P or H. After training, participants are then instructed to respond to the stimulus presented in the center of an array (e.g., SSPSS, SSMSS, targets underscored) and to disregard the “flanking” distractors (i.e., the Ss). Of all the flanker conditions, measures of interference such as RTs, error rates, and self-reported urges to err are lowest in the *Identical* condition, where flankers and targets are identical, as in SSSSS (Eriksen and Schultz, 1979; Morsella et al., 2009b). In this paradigm, it is well-established that interference is greater when distractors are associated with a response that is different from that of the target (*response interference*; e.g., SSPSS) than when distractors look different from targets but are associated with the same response (*perceptual interference*; e.g., SSMSS; van Veen et al., 2001; Morsella et al., 2009b). These findings, revealing that perceptual processes can automatically activate action plans, have been used as evidence for *continuous flow* (Eriksen and Schultz, 1979) and *cascade* (McClelland, 1979; Navarrete and Costa, 2004) models of perception-and-action (see discussion in Morsella, 2009; see, in this issue, Filevich and Haggard's treatment of the effects of unselected actions).

There are many other experimental paradigms that illuminate the study of consciousness and action control: the anti-saccade task (Hallett, 1978; Curtis and D'Esposito, 2009), the MacLeod and Dunbar object naming task (MacLeod and Dunbar, 1988), spatial compatibility tasks (e.g., the Simon task; Simon et al., 1970), response-effect compatibility paradigms (Kunde, 2001), the Posner attentional cuing task (1980), dual-task paradigms (Kahneman, 1973; Logan and Gordon, 2001), binocular rivalry (Alais and Blake, 2005), inattentional blindness (Raymond et al., 1992), covert priming paradigms (Bargh and Chartrand, 2000), the implicit association task (Greenwald et al., 1998), and the go/no go (Newman et al., 1985) and stop-signal tasks (Lappin and Eriksen, 1966; see, in this issue, articles by Anguera et al. and by Diefenbach et al.).

Evidence from these paradigms suggests that response interference stems from the automatic, “stimulus-triggered” activation of action plans (DeSoto et al., 2001), as if distractors automatically activate the associated action plans. Accordingly, psychophysiological research shows that, in response interference, competition involves simultaneous activation of the brain areas associated with the target- and distractor-related responses (DeSoto et al., 2001; Mattler, 2005). Complementary evidence has been obtained from a more micro level of analysis: The activity of the neurons in the motor cortex that, in the aggregate, yield a population code corresponding to one vs. another action (e.g., moving the arm left or right; Georgopoulos et al., 1983; Bagrat and Georgopoulos, 1999). This research reveals that individual neurons can be found to fire, not only for the target-related action (i.e., the intended actions), but also for distractor-related actions (Cisek and Kalaska, 2005). Interestingly, although neurons actively code distractor-related action plans, this activation does not appear to influence one's conscious awareness about ongoing action: One infers only that one's whole brain and musculature were concerned about executing the intended movement (see, in this issue, article by Filevich and Haggard). Research on automaticity (Puttemans et al., 2005) and on the consciously inaccessible neural mechanisms underlying action intentions (Libet, 2004) similarly reveal several sophisticated action-related processes that are unconscious.

Similarly, research on *mirror neurons* (Rizzolatti et al., 2008) has revealed that, when observing the actions of others, one is activating neural circuits that correspond to action planning, even though one may be motionless and utterly unconscious of these activations. This research also reveals that conscious percepts are intimately related to action control (James, 1890; Gibson, 1979; Llinás, 2002; Fuster, 2003). For example, Proffitt and colleagues (Proffitt et al., 2003; Witt et al., 2005) have shown that hills look steeper if one is carrying a heavy backpack or that objects appear closer when one is holding a tool that makes it easier to retrieve those objects (see also Firestone, 2013; Proffitt, 2013). For evidence regarding the role of functional knowledge in object identification, see Bub et al. (2003).

Additional evidence for unconsciously mediated action-related processing stems from the study of *efference binding* (Haggard et al., 2002a), which links perceptual processing to action/motor processing. This kind of stimulus-response binding allows one to learn to press a button when presented with a cue in a laboratory paradigm. Taylor and McCloskey (1990, 1996) demonstrated that, in a choice RT task, participants could select the correct motor response (one of two button presses) when confronted with subliminal stimuli (cf., Hallett, 2007). Unconscious efference binding also occurs in the case of reflexive responses to environmental stimuli, as in the *pain withdrawal reflex.* It is worth mentioning that, concerning unconscious integrations, the binding of perceptual information, known as *afference binding* (Morsella and Bargh, 2011) can also occur unconsciously, as is evident in intra- and inter-sensory illusions (e.g., the McGurk effect; McGurk and MacDonald, 1976). (The McGurk effect involves interactions between visual and auditory processes: An observer views a speaker mouthing “ga” while presented with the sound “ba.” Surprisingly, the observer is unaware of any intersensory interaction, perceiving only “da.”)

### Conscious aspects of action control

An appreciation of all that can transpire unconsciously during action control leads one to the following question. If so much in action control can be accomplished unconsciously, then what does consciousness contribute to action control? How and why is consciousness associated with some aspects of action control but not others?

When attempting to answer this question, one must consider that some aspects of action control do perturb consciousness strongly and reliably: (a) action-related mental imagery, (b) senses such as the *sense of agency* and *sense of effort*, and (c) action-related urges (e.g., arising under conditions of action conflict). We now discuss these under-explored conscious aspects of action control.

It has been demonstrated that the simultaneous activation of incompatible skeletomotor action plans, as when holding one's breath while underwater (where one is inclined to both inhale *and* not inhale) or suppressing a prepotent response in a response interference paradigm (see, in this issue, articles by Anguera et al., and by Lynn et al.), reliably influence consciousness (see quantitative review of evidence in Morsella et al., 2011). During such *conscious conflicts* (Morsella, 2005), a person experiences notable subjective “tuggings and pullings.” Lewin (1935), Freud (1938), and Miller (1959) studied the nature of these intra-psychic conflicts. Often, in such conflicts, the expression of undesired action plans can be suppressed, but the subjectively experienced action-related inclinations cannot be (Bargh and Morsella, 2008). For instance, a person can suppress dropping a painfully hot dish of porcelain, but cannot suppress the subjective urges to drop the expensive dish (Morsella, 2005). In this way, inclinations can be behaviorally suppressed but most often cannot be mentally suppressed (Bargh and Morsella, 2008). These conscious conflicts stand in contrast to (a) conflicts involving smooth muscle (e.g., involving the pupillary reflex; cf., Morsella et al., 2009a), and (b) perceptual conflicts, which tend to be unconscious, as in the case of ventriloquism and McGurk effects (McGurk and MacDonald, 1976). This pattern of results suggests that the skeletal muscle system (an effector given the special appellation, “voluntary muscle”) is intimately associated with conscious processing (see explanation in Morsella, 2005).

It should be noted that the interference paradigms mentioned above involve only punctate acts that are executed quickly (color naming and button pressing), placing minimal demands on *working memory* (WM). (See, in this issue, article by Anguera et al. and by Buchsbaum.) (WM has been defined as a temporary, capacity-limited storage system under attentional control that is used to intentionally hold, and manipulate, information in mind; Baddeley, 1986, 2007.) However, many of the conscious conflicts of everyday life—holding one's breath or gargling strong mouthwash for 30 sec—are not fleeting, short-lived events, but events that unfold over time and make demands on WM, by requiring one to hold in mind an action goal (e.g., not expelling mouthwash before 30 sec; Hommel and Elsner, 2009). In everyday life, many goal-directed actions are also guided by representations that are not triggered by external stimuli (Miller et al., 1960; Neisser, 1967). (This also occurs in the phenomenon of *prospective memory*; see McDaniel and Einstein, 2007.) Sustaining the activation of such internally-generated representations is an effortful process, requiring that top-down activation strengthen one representation (e.g., the target or action goal) over another (e.g., task-irrelevant goals; Gazzaley et al., 2005). Thus, many everyday acts of action control are actually instances of *WM-based action control*, in which a person effortfully holds an action goal in mind while attempting to overcome goal-irrelevant interference.

Theoretical developments have forwarded the notion that WM is intimately related to both action control and consciousness (LeDoux, 2008). This is evident in the title and contents of a recent treatise, *Working Memory, Thought, and Action* (Baddeley, 2007). Indeed, perhaps no mental operation is as consistently coupled with consciousness as is WM (LeDoux, 2008). When trying to hold in mind action-related information, a person's consciousness is consumed by this goal (James, 1890). For instance, when holding a to-be-dialed telephone number in mind (or when gargling with mouthwash for 30 sec), action-related mental imagery occupies one's consciousness during the delayed action phase. Similarly, before making an important toast (or, more dramatically, making the toast in a foreign and unmastered language), a person has conscious imagery regarding the words to be uttered, much as when an actor rehearses lines for an upcoming scene (see, in this issue, article by Buchsbaum). In this way, before an act, the mind is occupied with perceptual-like representations of what that act is to be, as James (1890) stated: “In perfectly simple voluntary acts there is nothing else in the mind but the kinesthetic idea… of what the act is to be” (p. 771). Thus, voluntary action control often occupies both WM and consciousness. Common experience suggests that, during the delay before action production, action-related imagery enters one's consciousness. The imagery is isomorphic in some ways with the overt action goal, especially in the case of “subvocalization” (Morsella and Bargh, 2010), which involves “talking in one's head” (Levelt, 1989). In subvocalizing, auditory imagery is isomorphic in some way with what would be uttered (Levelt, 1989; Baddeley, 2007; Morsella et al., 2009b; Morsella and Bargh, 2010).

In addition to conscious conflicts, urges, and WM-related conscious imagery is the sense of agency, another conscious aspect of action control. The sense of agency is based on the perception of the lawful correspondence between action intentions and action outcomes (Haggard and Clark, 2003; Wegner, 2003; Hommel, 2009). For example, if one has the intention of flexing one's finger or of saying “hello” and then one's finger happens to flex or one hears oneself utter “hello,” respectively, then one is likely to sense that one caused the action. This attribution is the outcome of conceptual processing (Synofzik et al., 2008a,b; Jeannerod, 2009) that takes into account information from various contextual factors (Wegner and Wheatley, 1999; Moore et al., 2009), including that of motor efference (Cole, 2007; Engbert et al., 2007; Tsakiris et al., 2007; Sato, 2009), proprioception (Balslev et al., 2007; Knoblich and Repp, 2009), and the perception of the real-world consequences of action intentions (Synofzik et al., 2009). This sense could be considered a form of *metacognition* (Dunlosky and Metcalfe, 2008).

By manipulating contextual factors, scores of experiments have demonstrated that subjects can be fooled into believing that they caused actions that were in fact caused by something else (Wegner, 2002). For example, when a participant's hand controls a computer-drawing device behind a screen such that the participant cannot see his or her hand in motion, the participant can be fooled into thinking (through false feedback on the computer display) that the hand intentionally moved in one direction when it actually moved in a slightly different direction (Fourneret and Jeannerod, 1998). With such techniques, participants in another study were tricked into believing that they could control the movements of stimuli on a computer screen through a phony brain-computer interface (Lynn et al., 2010). When intentions and outcomes mismatch, people are less likely to perceive actions as originating from the self (Wegner, 2002).

Most of these studies examine how agency is influenced by intention-outcome mismatches or illusory intention-outcome matches. There are several “comparator models” explaining how intention-outcome mismatches are detected and influence various levels of agency. Importantly, different theorists link the sense of agency and urges to different phases of the process (cf., Haggard, 2005, 2008; Berti and Pia, 2006; David et al., 2008). Complementing research on the sense of agency are investigations on the *sense of effort* during action control (Sherrington, 1900, 1906; Gandevia, 1982) and the sense of body ownership (e.g., in the rubber hand illusion; Botvinick and Cohen, 1998) and of actions generated toward the body (e.g., tickling-related illusions; Blakemore et al., 2000). Additionally, states described as *flow* (Csikszentmihalyi, 1990) and *effortless attention* (Bruya, 2010) have been associated with forms of action control. Moreover, theorists of the Würzburg School (e.g., Külpe, Ach, and Marbe) have discussed several, action-related *conscious attitudes*, including *doubt*, *hesitation*, *certainty*, and *will to enact a certain change in the world*.

We will now survey some less intuitive properties of action-related conscious processing. First, there is a peculiar property of voluntary action that appears to not be shared by other (e.g., involuntary) forms of action. For reasons unknown, in *intentional binding*, the perceived elapsed time between a voluntary action and its consequence is shorter than the actual time span (Haggard et al., 2002b), as if the two events were temporally attracted to each other. Thus, when striking a bell voluntarily, the experiences of striking the bell and of hearing the gong of the bell are perceived to occur more closely together in time than they actually did.

Another property of action-related consciousness arises in the paradigm of binocular rivalry (see Logothetis article). In this paradigm (see review in Alais and Blake, 2005), participants are first trained to respond in certain ways when presented with visual stimuli (e.g., to button-press when presented with the image of a house). After training, a different stimulus is presented to each eye (e.g., an image of a house to one eye and of a tree to the other). Surprisingly, the participant does not consciously perceive both objects (e.g., a tree overlapping a house), but responds as if perceiving only one object at a time (e.g., a house followed by a tree). During rivalry, the conscious percept is said to be “dominant,” and the unconscious percept is said to be “suppressed.”

The mind's process of switching dominance between each eye can be manipulated in interesting ways. Maruya et al. (2007) demonstrated that voluntary action can influence which percept enters awareness: The object that moved in synchrony with participants' voluntary movements of a computer mouse was dominant for a longer period of time and suppressed for a shorter period of time. Rivalry stimuli consisted of a radial grating (resembling the pattern on a dart board) and a rotating sphere that was transparent and defined solely by dots. Prior to test, participants learned to move a computer mouse in a continuous left-to-right motion. Participants later performed this motion under conditions of rivalry. Maruya et al. (2007) concluded, “conflict between two incompatible visual stimuli tends to be resolved in favor of a stimulus that is under motor control of the observer viewing that stimulus” (p. 1096), revealing “a strong link between action and perception” (p. 1090). This finding is consistent with that of Wohlschläger (2000), who reported that, while perceiving a perceptually bistable apparent rotation of an object, participants were more likely to perceive the object as rotating in the direction in which they happened to be rotating a knob (Repp and Knoblich, 2007), a case of *perceptual resonance* (Wohlschläger, 2000; Schütz-Bosbach and Prinz, 2007). Consistent with the finding by Maruya et al. (2007), Doesburg et al. (2009) found in a psychophysiological study that it is only during the dominant percept that perceptual processing associated with the percept is coupled with motor-related processes in frontal cortex. (Additional evidence stems from a recent study showing that entry of any kind may require a top-down signal from frontal cortex; Boly et al., 2011; Panagiotaropoulos et al., 2012.)

Perceptual resonance, and the voluntary control of action, can be explained by *ideomotor theory* (Lotze, 1852; Harleß, 1861; James, 1890; Greenwald, 1970; Hommel et al., 2001; Hommel, 2009; Hommel and Elsner, 2009). When popularizing this theory, William James (1890) proposed that the mere thoughts of actions produce impulses that, if not curbed or controlled by thoughts of incompatible actions, result in the performance of the imagined actions (see Marien et al., this issue). From this view, activating the perceptual effects of an action leads to the corresponding action—effortlessly and without awareness of the motor programs involved (Gray, 1995; Kunde, 2004). The representations guiding action production tend to be perceptual-like images of action outcomes (Hommel, 2009), which are based on memories of prior action outcomes (see, in this issue, Marien et al. for role of reward in ideomotor learning). Consistent with ideomotor theory, during conflicts such as those of the Stroop task, it is perceptual-like representations that are activated to guide action (Enger and Hirsch, 2005).

Because action/motor processes are largely unconscious (Grossberg, 1999; Goodale and Milner, 2004; Gray, 2004), the entry into consciousness of content is influenced most by perceptual-based (and not action-based) events and processes (e.g., priming by perceptual representations; Müller, 1843; James, 1890; Gray, 2004; Morsella and Bargh, 2010). [See brain stimulation evidence in Desmurget et al. (2009).] Hence, few conscious contents should arise from what can be construed as “pure” action-related processes (should there be such a thing; cf., Hommel, 2009). Thus, entry from action in Maruya et al. (2007) might be the result of the more “perceptual” aspects of action production, such as perceptual-like *action effect* representations (or “Effektbild”; Harleß, 1861) or corollary discharges from action plans (Gray, 2004). From this standpoint, though perception and action are intimately related and may even share the same representational format, as in “common code” models of perception-and-action (Hommel, 2009), when it comes to phenomenology, consciousness is most influenced by what has traditionally been regarded as the perceptual end of the perception-action cycle (Neisser, 1976; Gray, 1995). Accordingly, research by Wohlschläger (2000) and by ideomotor theorists (e.g., Hommel, 2009) suggests that action-based effects on awareness such as perceptual resonance require, not only perturbation of the sensorium, but dimensional overlap (e.g., shared spatial dimensions) between actions and percepts (cf., Knuf et al., 2001; Schütz-Bosbach and Prinz, 2007).

As noted, some ideomotor models propose that perceptual action effects and action codes share the same representational format, hence the description of some ideomotor accounts as common code theories of perception-and-action (Hommel, 2009). Such common code perspectives resemble mirror neuron approaches (Rizzolatti et al., 2008) and motor theories of speech perception (Liberman and Mattingly, 1985). (For a treatment of action simulation, see, in this issue, Springer et al.) Similarly, speaking about the interconnection between perception and action, Sperry (1952) proposed that the phenomenal percept (e.g., the shape of a banana) is more isomorphic with its related action plans (grabbing or drawing the banana) than with its sensory input (the proximal stimulus on the retina). [For contemporary treatments regarding how action influences the nature of conscious percepts, see Gray (1995), Hochberg (1998), O'Regan and Noë (2001), and Humphreys (2013).]

With great influence, Gibson (1979) too proposed an “ecological theory” of perception in which perception is intimately related to action, but, unlike ideomotor theory and common code approaches, Gibson's approach is strictly non-representational in that all the information necessary for action was provided and contained by the environment. For a treatment regarding the difference between ecological and representational (“cognitive”) theories of action, see Hommel et al. (2001). See Sheerer (1984) and Markman (1999) for reviews of the shortcomings of approaches in which the nature of percepts or, more generally, representations, is constituted in part by motor processing, as in “peripheralist,” “motor,” “embodied,” “efferent,” and “reafferent” theories of thought (e.g., Münsterberg, 1891; Watson, 1924; Washburn, 1928; Held and Rekosh, 1963; McGuigan, 1966; Festinger et al., 1967; Hebb, 1968; see discussion of embodied approaches in Deifenbach et al., this issue; see relevant article by Jordan, in this issue).

### Conclusion to the introduction of the special issue on consciousness and action control

Our survey and the following articles reveal that one of the primary reasons to study consciousness by way of action control is that the contrast between conscious and unconscious processes is easy to appreciate from an action-based standpoint. It is important to consider that, though it is far from trivial to demonstrate unconscious perceptual processing—a controversial phenomenon whose study often requires neuroimaging and sophisticated techniques (e.g., perceptual priming)—even the most cursory examination of action phenomena reveals that, in the nervous system, there is the distinction of processes that are *consciously mediated* (e.g., voluntary action) and *unconsciously mediated* (e.g., reflexes, peristalsis, and aspects of motor control). Stumbling upon this contrast between conscious and unconscious processes is not only uncontroversial in the study of action but is inevitable. In addition, it is more experimentally tractable to study the relationship between action and consciousness than that between attention and consciousness (the traditional approach; cf., Baars, 1997), because in the former there is less likelihood of conflating conscious and attentional processes (cf., Hamker, 2003), a recurring problem in consciousness research (Baars, 1997; Maruya et al., 2007). Last, what Sperry noted in 1952 about action is still true: *The outputs of a system reveal more about the inner workings of the system than do the inputs to the system*. As the cardinal “output” of the nervous system (Morsella and Bargh, 2010), action thus provides the investigator with a unique portal to illuminate the most elusive of central processes, consciousness.

### Conflict of interest statement

The authors declare that the research was conducted in the absence of any commercial or financial relationships that could be construed as a potential conflict of interest.

## Footnotes

^1^Consider that the artificial heart is very different from its natural counterpart and that the difference between human locomotion and artificial locomotion is a stark one—that between legs versus wheels.

^2^It is important to appreciate that, even in the early Twentieth Century, in the field of psychiatry (which was at that time independent from psychophysics and other forms of academic psychology), the student of the mind realized that in the nervous system there are processes that are consciously mediated and those that are unconsciously mediated, as discussed at length and with great insight by the psychiatrist Bleuler (1924).

^3^The following is based in part on reviews of the literature presented in Morsella and Bargh (2011); Morsella et al. (2011) and Hubbard et al. (2013).
